# Fracture of Femur—An Appeal

**Published:** 1892-07

**Authors:** T. Dwight Stow

**Affiliations:** Mexico, N. Y.; Bureau of Surgery, I. H. A.


### FRACTURE OF THE FEMUR—AN APPEAL.

T. Dwight Stow, M. D., Mexico, N. Y.

(Bureau of Surgery, I. H. A.)

Fracture of the femur, though not uncommon, is not as common as fracture of the tibia, humerus, radius, or ulna. But it is a more serious accident than either of the other fractures mentioned—at least in respect of easy and nice adjustment. Contrary to the rule, with the long bones, the femur is most often broken at the upper end of its middle third; next in order at the neck and without the capsule; least often in its lowest third. No age is exempt, but persons between the ages of fifty and eighty are more likely to sustain the injury, the reparative power diminishing as age advances after fifty; being in healthy subjects at its maximum at forty or forty-five years. Children between five and fifteen years recover soonest, and with the likelihood of less deformity. Force applied perpendicular to the long axis of the shaft generally produces fracture at the middle third. A fall from considerable height landing on the knees or feet, generally results in a fracture through the condyles, vertically, or in the fracture of the neck.

Surgeon Hamiliton, in his work on *Fractures and Dislocations*, cites one hundred and fifty-six fractures of the femur, not including gunshot, of which sixty-three belong to the upper third; sixty-seven to the middle third, and twenty-six to the lower third. Fractures of the neck of femur are more common among people above seventy, though occasionally fracture happens to persons much younger. Epiphyseal separation is the most common kind among children.

Females seem to be more prone to fractures of the neck of femur than males; probably because they usually fall on ice or slippery pavements, striking on the buttocks heavily, or on the trochanters. Rarely fractures of the femur are caused by undue muscular action. Instances are recorded of such fracture occurring in bed, but generally among the aged.

Of the several varieties of femoral fracture, simple, transverse, compound, or comminuted, each has its characteristics to be considered and treated according to its peculiarities and the tact of the surgeon. But there are at least four steps to be taken at all times and on every occasion in order to insure success—they are:

The diagnosis.

The reduction and apposition of the broken bone.

The confinement and fixity of limb.

Counter extension.

It is often necessary to anæsthetize the patient in order to successfully diagnose the case and reduce the fracture, and this is particularly true if the patient be very fat, or muscular, or nervous, and the fracture be at the neck of femur.

It is not enough to know that the patient had a fall or blow in this or that direction; preternatural mobility, deformity, shortening, crepitus, pain, and swelling; but one needs to recognize the *location* and *kind* of fracture, whether simple, comminuted, transverse, or oblique. The surgeon needs coolness, caution, good tactile qualities, and no haste.

The patient ought not to lie on anything more flexible than a thick, well-stuffed hair, husk, or cotton mattress. If the bed be wide, he or she should be brought well to the front of the bed. For convenience, comfort, and safety to the patient and limb, the bed should be provided with a trap for purposes of defecation and urination. The hips should be level and kept so, and the shoulders may be raised at time of reduction to an angle of forty-five degrees. All should be made ready for reduction before anæsthesia.

### Reduction.

Wash the limb, and if the surgeon chooses to make necessary extension by means of adhesive strips applied to leg, shave the leg of all hair and apply the strips, securing them by a roller or by other strips passed about the limb. A better way is to apply a plaster of Paris bandage to the leg from toes to knee, weaving in some strong webbing about an inch wide, down the sides of the leg; for this makes the best extending appliance—though a soft shoe worn by the patient, or a stiff buckskin upper—may be substituted.

Next, an upper and posterior Ahl's or Levis's femoral splint should be applied over the seat of fracture, always excepting fracture of the neck of femur. These splints should be padded with clean white sheet-wadding, or absorbent cotton, or soft flannel. Now straighten the limb its full length until the internal malleoli meet, centre to centre; they touching a line drawn from centre of manubrium through the symphysis pubis downward. Adjust the fractured ends; and if the fracture be compound, leave an opening sufficiently large for inspection and drainage, and dressing. First, confine the splints to thigh, by means of adhesive plaster passed around the entire limb over the splints, confining them snugly to the same. Over the splints, apply a roller from knee to the gluteal crease, or as near that point as is possible. I often apply over the splints a russet leather buckskin-lined case, having from four to five billets and buckles, so that the case can be readily tightened as the swelling of limb diminishes. At the outer and upward end of this case, a ring should be attached, from which extension upward may be made.

The next step to be taken is to place the entire limb on a mattress about one and a half inches thick; as long as the entire limb, wedge-shaped, wide as the greatest diameter of the thigh at the upper end, and about four inches wide immediately under the ankle. This mattress should rest on a splint of board about one-half inch thick and long enough to reach from the crest of ilium to the heel of fractured limb, and a little beyond. This bed splint should have a side piece of nearly the same length, five inches wide—more or less—according to diameter of thigh, and three, or less inches wide at the lower end. This should be three-eighths or one-half inch thick, and screwed to the edge of bed splint—the width of this splint should be calculated from the surface of the mattress, before mentioned. The limb may be tied to this splint at two points above and two points below the knee, to keep it in position. Counter extension from the webbing woven into the plaster of Paris or starched bandage, may be made on a line corresponding with the centre of the limb in its long axis, and should be made over a pulley. A bag of sand or any other weight as convenient, should be maintained daily, for at least six weeks; but after the third week the weight may be taken off a few minutes at a time, twice daily, to rest the patient. From five to ten pounds or even twelve pounds may be used, according to age and strength of patient. I have mentioned the "plaster of Paris" bandage as the best that can be used from which to make extension. But, it occasionally transpires that erysipelatic inflammation of the entire limb occurs, hazardous to the patient, perplexing to the surgeon, preventing any compression of the limb by adhesive plaster or other bandaging. I have had several such cases, not only of fracture of the femur, but of the tibia, that could be managed only by putting the limb in a fracture-box, packing it with baked sifted pine sawdust—the best packing out—but the skin may be protected from the sawdust by first laying upon it thoroughly boiled and sterilized cheese cloth. Afterward, counter extension may be made partly from dorsum of foot and heel, and the bottom of fracture-box; superior extension, by a broad but soft perineal bandage.

The length of time requisite to safe and complete union, varies from six to ten weeks, according to age and general condition of patient.

An oblique, or comminuted fracture requires more care, more time as a rule.

Formerly, in the application of splints, it was deemed absolutely requisite, to get the splints long enough to occupy the entire space between the condyles, and to press against them. Now it is not demanded. Indeed, in most cases, such practice would give rise to much irritation and inflammation of skin; while in fracture extending into the knee-joint, it would be positively injurious and risky.

### REMARKS ON SPECIAL CASES.

In caring for fracture of neck of femur within or without the capsular ligament, the mattress on which the patient rests should be supported on two or three matched boards, battened at each end. One of the neatest fracture and general use beds is the Miller: one long, straight, well-padded splint, reaching from the axilla to some four inches beyond sole of feet to steady body and limb, with some bags placed at such points as are necessary to keep the limb in place, and prevent undue rotation are, with the requisite bandaging and counter-extension, enough. Aged and infirm persons ought not to be subjected to the worry, irritation, and trouble of confinement in splints.

Sir Ashley Cooper's instruction on this point is most valuable: "Place the patient on an easy unyielding bed; draw the knee well up and place a pillow under it; let him rest until all pain, heat and swelling entirely disappear, and he can get about on crutches. False joint may follow; let it."

Oblique or comminuted fracture at or near centre of the shaft, requires mainly—after reduction—sufficient counter extension, and after the heat, swelling and pain abate as close compression of the limb at point of fracture as good circulation will admit of.

Fracture of femur extending into the knee is best managed by placing the limb on a well-padded Day or Amesbury splint. If the condyles be split, quite large but not hard pads should be placed each side of the tuberosities, and kept snugly in place by a few turns of woolen roller.

Compound fracture should be treated in the ordinary manner, but with a view to aseptic dressing, drainage, etc.

Let it be borne in mind—and the patient should be informed of this—that under the best of management, shortening of the limb is likely to occur to a greater or less extent. This is emphatically true of comminuted and oblique fracture of the shaft, the shortening ranging from three-eighths to two and one-half inches. Strong muscular men are most likely to have such shortening and deformity; children least likely to. Among the reasons explanatory of this are: non-union, preternatural softness or too rapid absorption of the provisional callus; endo-ossic inflammation; hemorrhage into the Haversian canals; too early use of limb; unusual muscular contraction, especially during sleep; carelessness of patient or practitioner.

As a rule a liberal regimen is admissible, but not too amylaceous.

Much litigation, much loss of prestige to the surgeon has followed the treatment of several varieties of femoral fracture. Many surgeons have been subjected to loss of fee or to expensive suits at law, and heavily "mulcted," through their own ignorance or carelessness, or on account of public ignorance and misconception of the nature of fracture and the surgeon's responsibility. There is also a great amount of moral obliquity shown by many of the surgeons of the old school, when summoned as witnesses against members of the homœopathic fraternity; some going so far as to say: "They know nothing of anatomy, surgery, etc.; anything to cripple a follower of Hahnemann. If surgeons of our school are needed, and we practically admit they are, by stoutly indorsing resolutions second and third of the late Declaration of Principles, and by establishing for this association a Bureau of Surgery, then it is of equal importance to sustain them, and it. Most of our practitioners are educated in the department of Anatomy and Surgery, and thus qualified to know and defend good surgery and good surgeons. We who have experiences in these matters, know that the pain of injuries, the pain after operations, symptomatic fever, exhaustion after hemorrhage, inflammatory conditions, the neuralgia of diseased ovaries and of the womb, or of diseased testicles, cord, prostate, etc., and suppurative processes terminating in abscess, are generally, wonderfully controlled by the indicated and dynamized remedy. We are not afraid to challange comparison with the best old-school therapeuticians in this respect. We do not, however, claim superiority as *operators;* that would be too much; though in this respect—with becoming modesty—we trust our best surgeons are not far behind. If any are somewhat curious to learn about what is the average of old-school treatment of surgical cases, we would cite the reports on pages 100 to 106 of the May number of *International Journal of Surgery*—1891. "A case of Cæsarean Section; another of Cholecystectomy." In the first case, it is a wonder the patient recovered; in the second case it is no wonder she died. To rescue humanity from such hazardous treatment and present the advantage of pure Homœopathy, in surgical cases, is part of our mission. If in this you agree with me, let me *urge you* to stand by the Bureau of Surgery, making its interest your own. Supply it with clinical reports and listen to its papers. Moreover, as far as it be possible, send or otherwise furnish those of your number who by reason of special study, of operative tact, and outfit are prepared to operate such cases as coming under your control or advice can be prevented from passing into old-school hands. In my humble estimation, a serious fault of our practitioners is: they incline to neglect surgery, and thus cripple their own usefulness, and limit the spread of our ideas. Outside the cities, there is a paucity of homœopathic surgeons. Every county ought to have at least three good surgeons, who would agree to assist each other. This would strengthen our cause, and greatly increase its popularity.

